# Tobacco industry now openly admits that it is aware that cigarettes are harmful

**DOI:** 10.18332/tid/113509

**Published:** 2019-11-08

**Authors:** Hedley K. Quintana, Reina Roa

**Affiliations:** 1 Gorgas Memorial Institute for Health Studies, Panama City, Panama; 2 Tobacco Control Focal Point, Ministry of Health, Panama City, Panama

**Keywords:** law, WHO FCTC, criminal, law, vaping advertising, e-cigarette advertising

**Dear Editor,**

Tobacco products are responsible for a large number of deaths and disability burden^1^. The tobacco industry (TI) had not openly made a statement regarding the harms associated with the products they sell, until now. TI is now selling new tobacco products as part of a ‘harm reduction’ strategy. These new tobacco products include e-cigarettes, vaping and similar devices. TI states that these new products are ‘safer’ than the ‘conventional cigarettes’ marketed by them. Health authorities have been concerned about the dangerous health effects of tobacco products and their message has been consistent over time. However, Philip Morris and British American Tobacco now admit that ‘conventional cigarettes’ are associated with health risks^2,3^ in order to promote the new products they sell, the health risks associated with cigarette smoking are given on both archived TI websites. These risks include secondhand smoke, cardiovascular and lung diseases, as well as pregnancy-associated complications. Although Japanese International Tobacco and Imperial Brands also offer similar new products, which they state have ‘lower risks’^4,5^, they do not indicate what these risks are. In our view, these recent statements by the TI represent an open admission that they are fully aware of the harm of their products, but despite this they continue to sell them. The statements by the tobacco industry represent clear evidence of its mens rea regarding possible criminal cases for tobacco-associated deaths, disability and violations of human rights. Furthermore, why should we believe TI statements about the safety of their new tobacco products if they have lied about the cigarettes they also sell.

## CONFLICTS OF INTEREST

The authors have completed and submitted the ICMJE Form for Disclosure of Potential Conflicts of Interest and none was reported.
